# CCNG1 (Cyclin G1) regulation by mutant‐P53 via induction of Notch3 expression promotes high‐grade serous ovarian cancer (HGSOC) tumorigenesis and progression

**DOI:** 10.1002/cam4.1812

**Published:** 2018-12-18

**Authors:** Ying Xu, Qing Zhang, Chunying Miao, Samina Dongol, Yinuo Li, Chenjuan Jin, Ruifeng Dong, Yingwei Li, Xingsheng Yang, Beihua Kong

**Affiliations:** ^1^ Department of Obstetrics and Gynecology, Qilu Hospital Shandong University Ji'nan China; ^2^ Gynecology Oncology Key Laboratory, Qilu Hospital Shandong University Ji'nan China

**Keywords:** Cyclin G1, high‐grade serous ovarian cancer, metastasis, Notch3, P53mt, prognosis

## Abstract

TP53 mutation is considerably common in advanced high‐grade serous ovarian cancer (HGSOC) and significantly associated with a poor prognosis. In this study, we investigated the role of Cyclin G1 (CCNG1), a target gene of wild‐type *TP53* (P53wt), in HGSOC and the possible regulatory mechanism between *TP53* mutant (P53mt) and *CCNG1* in the progression of HGSOC. High expression level of CCNG1 was found in 61.3% of HGSOC tissues and only 18.2% in fimbriae of fallopian tubes. Additionally, overexpression of CCNG1 was significantly associated with a shorter overall survival (*P *< 0.0001) and progression‐free survival (*P < *0.0004) in HGSOC patients. In vitro, CCNG1 promoted both tumor cell motility by inducing epithelial‐mesenchymal transition (EMT) and resistance to cisplatin (CDDP). In vivo, knockdown expression of CCNG1 inhibited cancer metastasis. Furthermore, P53mt increased the expression of CCNG1 by regulating Notch3 expression, and a positive correlation between CCNG1 and Notch3 protein expression was observed by Immunohistochemistry (IHC) (*r* = 0.39, *P*: 0.01528). In conclusion, the activation of P53mt‐Notch3‐CCNG1 pathway was responsible for tumor progression to advanced disease with correlation with worse prognosis in patients with HGSOC. These data suggest a possible molecular mechanism of disease and highlights CCNG1’s potential role as a therapeutic target in HGSOC.

## 1 INTRODUCTION

Epithelial ovarian cancer (EOC) is the most lethal gynecologic malignancy and more than 240 000 women develop EOC worldwide each year.^1^, ^2^ Most EOC patients are initially asymptomatic and ultimately diagnosed at an advanced stage.^2^, ^3^ Four main histologic subtypes of EOC exist: serous, endometrioid, mucinous, and clear cell types; and high‐grade serous ovarian cancer (HGSOC) accounts for nearly 70% of all EOC.^4^, ^5^ Despite a combination of treatment methods, including chemotherapy and targeted therapy, more than 90% of the patients with advanced disease develop recurrence, resulting in a 5‐year survival rate of <40%.^7^ Metastasis and cisplatin (CDDP) resistance are two of the most challenging obstacles in successful treatment of HGSOC.^8^, ^9^ Understanding the molecular mechanisms of both HGSOC metastasis and chemotherapy resistance are of extreme importance and could potentially improve patient survival.

The tumor suppressor gene*TP53* encodes for protein P53 and is the most frequently mutated gene in human cancer.^10^ Emerging data suggest that a mutant protein P53 (P53mt) is associated with genomic instability, aberrant cell cycling, invasion, metastasis, and drug resistance.^10^, ^11^ It is known that P53 mutation occurs in almost all HGSOC (96%).^11^ Kuhn et al^13^ additionally reported that missense mutations of *TP53* were observed in 61% of serous tubal intraepithelial carcinomas (STIC), which is regarded as the precursor of HGSOC. Thus, it is reasonable to infer that TP53 mutation may act as a driving event in the development of HGSOC.

Cyclin G1 (CCNG1) is a Cyclin G family protein that both positively and negatively regulates cell growth.^14^ Although the precise function of CCNG1 remains unclear, accumulating evidence has shown that CCNG1 is abnormally expressed in many types of malignant cancers, such as EOC, hepatocellular carcinoma, and lung carcinoma.^15^, ^16^ Some studies have found that CCNG1 can act as a transcriptional target of P53, suggesting that CCNG1 may serve a significant role in the poor prognosis of HGSOC.^16^, ^18^, ^19^

Notch signaling has been implicated in various tumor processes, including cell differentiation, metastasis, proliferation, and drug resistance.^20^ In mammalian cells, this pathway consists of five transmembrane Notch ligands (Jagged‐1, Jagged‐2, Delta‐like ligand (DLL) 1, DLL3, and DLL4) and four Notch receptors (Notch1‐4).^21^ It has been reported that Notch pathway alterations are prevalent in HGSOC. Notch3 is overexpressed in approximately two‐thirds of HGSOC, making it a potential candidate for targeted therapy.^22^, ^23^ The relationship between the Notch3 pathway and the other aforementioned cell regulatory pathways is not well established. In this study, we explored the role of CCNG1 in HGSOC tumorigenesis, as well as the regulatory mechanisms between CCNG1 and P53mt‐Notch3 pathway.

## 2 MATERIALS AND METHODS

### 2.1 Tissue samples

Tissue samples were collected from 266 patients with HGSOC who underwent surgical resection at the Qilu Hospital of Shandong University between 2005 and 2013, and Peking Union Medical College Hospital between 2003 and 2009. The age range was 36‐78 years (median: 55 years). All HGSOC patients were diagnosed based on clinical protocols, and none received neoadjuvant chemotherapy or immunotherapy. Of 266 patients, 51 patients were diagnosed at early stages (stages I‐II) and 214 patients were diagnosed at advanced stages (stages III‐IV). The mean follow‐up period was 42.2 months (ranging from 2 to 130 months). Normal control tissues, 55 fimbriae of the fallopian tube (FTE), were collected from patients who underwent surgical resection with benign neoplasms at the Qilu Hospital of Shandong University. For inclusion within the study, all FTE specimens were evaluated with TP53 immunohistochemical staining to ensure they were not precancerous (Figure S6).

### 2.2 Immunohistochemistry (IHC)

The 266 HGSOC and 55 FTE tissues were fixed in formalin for 24 hours Tissue sections (4 μm thick) were obtained, deparaffinized in xylene for 15 minutes, and rehydrated in a graded series of ethanol. Antigen retrieval was performed by microwave irradiation at 98°C for 10 minutes in 10 mmol/L EDTA buffer (pH 8.0 for CCNG1) or 10 mmol/L citrate buffer (pH 6.0 for Notch3). Endogenous peroxidase activity and nonspecific binding were blocked separately with 3% hydrogen peroxide in methanol for 15 minutes and donkey serum for 30 minutes. The following panel of antibodies was used: diluted anti‐Cyclin G1 antibody (dilution 1:100; Abcam, Cambridge, MA, USA; ab49274) and anti‐Notch3‐antibody (dilution 1:100; Santa Cruz, CA, USA; sc‐5593) at 4°C for 12 hours. Antibody dilution buffers were purchased from Beyotime (China, P0023A).

Staining was visualized with a VENTANA iScan scanning system, which has an automated turret with four objectives for optical scanning at 4X, 10X, 20X, and 40X magnifications. In order to evaluate the staining intensity in the cell nuclei（CCNG1）and cytoplasm（Notch3), four scores were defined [0, negative (−); 1, weak(+); 2, medium (++); 3, strong(+++)]. Also, the proportion of ovarian cancer and FTE epithelial cells was scored from 0 to 4 (0, 0%; 1, 1%‐25%; 2, 26%‐50%; 3, 51%‐75%; 4, 76%‐100%) on each section. Both CCNG1 and Notch3 expression were interpreted and graded according to the product‐sum of staining intensity and proportion of staining scores. The samples were considered high expression if the product‐sum was five or greater and low expression if it was <5.

### 2.3 Plasmid and transfection

The full lengths of CCNG1 (Table 1) were synthesized by Biosune (Shanghai, China) and inserted into Sgf1/MIu1 sites of a PLenti‐C‐Myc/DDKvector (OriGene, 10069). PLenti‐C‐Myc/DDK vector was designated as the mock control. The vector pCMV‐p53mt135 (631922) was bought from Conetech, USA.

Plasmid GV141‐NICD3 (Notch3 intracellular domain plasmid; amino acids 1663 to 2312) was constructed by cloning the NICD3‐coding region (NM_000435‐P1) to GV141 vector (Genechem, CON106). GV141 null vector was designated as the mock control. PCR primers used for amplification of the full‐length cDNAs are shown in Table 1. Ultimately, shRNAs were used to knockdown CCNG1 and Notch3 pathways. Empty pLKO.1 vectors (Addgene, 10878) were used as controls (shRNA sequences displayed in Table 2).

**Table 1:** PCR primers used for amplification of the full‐length cDNAs

Gene	Sequence (5′‐3′)
CCNG1‐F	GAGGCGATCGCCATGATAGAGGTACTGACAACAACT
CCNG1‐R	GCGACGCGTTTAAGGGACCATTTCAGGAATTG
Nothc3‐F	ACGGGCCCTCTAGACTCGAGCGCCACCATGGTGGCCCGGCGCAAGCGCGAG
Notch3‐R	TTGGTACCGAGCTCGGATCCACTTCCGGCTGGGGCCCCAGCTG

**Table 2:** ShRNA sequences

Gene	ShRNA sequences
CCNG1	CCGGCCAAATGTTCAGAAGTTGAAACTCGAGTTTCAACTTCTGAACATTTGGTTTTTG
Notch3	CCGGTTTGTAACGTGGAGATCAATGCTCGAGCATTGATCTCCACGTTACAAATTTTTG
P53	CCGGCGGCGCACAGAGGAAGAGAATCTCGAGATTCTCTTCCTCTGTGCGCCGTTTTT

For stable infection, Lentivirus expressing CCNG1 and Plko.1‐shRNA (CCNG1, Notch3, P53 proteins), packaged with psPAX2 (Addgene, 12260) and pMD2G (Addgene, 12259), were produced in HEK293T cells with Lipofectamine 2000 (Invitrogen, 11668019) according to a protocol (Appendix S1). After transfection by Lentivirus for 24 hours, the cells were selected in medium containing 2 μg/mL puromycin (Merck Millipore; Burlington, MA, USA) for two weeks. Stable expression cells were obtained and expanded for further studies.

### 2.4 RNA extraction and real‐time polymerase chain reaction (RT‐PCR)

Total RNA (500 ng/μL) was extracted from tissue samples or cultured cells by using TRIzol reagent (Invitrogen; Carlsbad, CA, USA). Then, mRNAs (500 ng) were reverse‐transcribed into cDNA by PrimeScript™ 1st Strand cDNA Synthesis Kit (Takara, Japan) according to the manufacturer's protocol. After cDNA mixed with SYBR Green (Takara, Japan), RT‐PCR was conducted using ABI 7900HT Fast Real‐Time PCR System (Applied Biosystems; Foster City, CA, USA) with the housekeeping gene glyceraldehyde‐3‐phosphate dehydrogenase (*GAPDH*) as an internal control. PCR primers were designed according to the GeneBank sequences (Table 3). The comparative threshold cycle method: 2^‐ΔΔCt^ was used to calculate the relative gene expression level (amount of target gene normalized to endogenous control gene). The range of the obtained Ct values was 15‐35.

**Table 3:** Primer sequences for RT‐PCR

Gene	Primers sequence (5′‐3′)	Annealing T (°C)
CCNG1‐F	AATGAAGGTACAGCCCAAGCA	63
CCNG1‐R	GCTTTGACTTTCCAACACACC	
β‐actin‐F	GAGGCACTCTTCCAGCCTTC	55
β‐actin‐R	GGATGTCCACGTCACATTC	
Notch3‐F	TCTCAGACTGGTCCGAATCCAC	57
Notch3‐R	CCAAGATCTAAGAACTGACGAGCG	
P53‐F	TGAAGTCTCATGGAAGCCAGC	54
P53‐R	GCTCTTTTTCACCCATCTACAG	

### 2.5 Cell culture and reagents

Human ovarian cancer cell line A2780 (Procell, China, CL‐0013) and HO8910 (Procell, China, CL‐0113) were cultured in RPMI 1640 culture medium and SKOV3 (ATCC, USA, HBT‐77) in McCoy's 5A medium, supplemented with 10% fetal bovine serum (FBS, Gibco). OVCAR3 (ATCC, USA, HTB‐161) was cultured in RPMI1640 with 20% FBS (Gibco, USA). HEK293T (ATCC, USA, CRL‐3216) was cultured in DMEM culture medium with 10% FBS (Gibco, USA). All cells were grown at 37°C under 5% CO_2_. Cisplatin was purchased from SIGMA ALDRICH (P4394, USA). RNA was extracted from each cell types three times, thus three relative gene expression levels of the target gene from each sample were obtained.

### 2.6 Cell migration and invasion assay

A number of 1.5 × 10^5^ cells were resuspended in FBS‐free medium and seeded into the top chambers of Transwell® inserts (FER 353097, 24‐well, 8 μm pore size; BD Bioscience) and Transwell® inserts (35448024‐well, 8 μm pore size; Corning); 700 μL medium supplemented with 20% FBS was added into the bottom chambers as a chemoattractant. After 12‐24 hours incubation, we wiped away the cells on the upper surface of the membrane. Next, the cells on the lower surface of the membrane were washed with phosphate buffer saline (PBS), fixed with methanol, and last stained with 0.1% crystal violet to quantify the extent of migration and invasion.

### 2.7 Western blotting (WB)

Proteins were extracted from cultured cells, which were treated with RIPA Lysis Buffer ((Beyotime, P0013C)) and 1% PMFS. A total of 30 μg protein per well were separated by SDS‐PAGE (5% stacking gel and 10% separation gel), transferred to polyvinylidene difluoride membranes (0.2 μm Millipore ISEQ00010) by BIO‐RAD Trans‐blot (15 V 90 minutes), blocking with 5% skimmed milk (232100, BD USA) solution for 1 hour at 25°C and then treated with the primary antibodies overnight at 4°C for 16 hours On the following day, the cells were rinsed with TBST and incubated with secondary antibodies conjugated with horseradish peroxidase at 25°C for 1 hour The monoclonal antibodies used in this study are as follows: mouse antihuman Cyclin G1 antibody (1:200 dilution; Abcam, Cambridge, MA, USA; ab49274), rabbit antihuman Notch3 antibody (1:1000 dilution, Santa Cruz:sc‐5593), mouse antihuman p53 antibody (1:1000 dilution; Dako Products, Santa Clara, CA; ABCA0332729), rabbit antihuman N‐CAD antibody (1:1000 dilution, CST:13116), rabbit antihuman E‐CAD antibody (1:1000 dilution, CST:3195), rabbit antihuman slug antibody (1:1000 dilution, CST), rabbit antihuman snail antibody (1:1000 dilution, CST:3879), mouse antihuman β‐actin antibody (1:1000 dilution, CST:3700), and peroxidase‐conjugated secondary antibody (1:5000 dilution Sigma A0545, A9044). The bands were detected by an Imagequant LAS 4000 (GE Healthcare) system with the Western Lighting Plus‐ECL (PerkinElmer, 203‐17201). The β‐actin was used as a loading control. Gray level was analyzed by image J. Proteins were obtained from each cell types for three times, and three gray values of bands were ascertained. Unprocessed blots are shown in Figure [Link], [Link], [Link], [Link], [Link].

### 2.8 Cell viability detection

A total of 2500 cells were seeded in 96‐well plates, and after adhesion to the plate, they were exposed to cisplatin at various final concentrations: 0, 1.25, 2.5, 5, 10, or 20 μg/mL for 24 hours Each concentration was repeated in quintuplicate wells. After incubation with 20μL of 5 mg/mL 3‐(4, 5)‐dimethylthiazol (−zyl)‐3, 5‐diphenyltetrazolium bromide (MTT; China) for 5 hours, the medium was exchanged with 100 μL of DMSO. And then cell viability was measured using the Varioskan Flash microplate reader (ThermoScientific; Waltham, MA, USA). The experiment was performed in triplicate.

### 2.9 In vivo nude mouse metastasis assay

Plko.1‐shCCNG1–transfected A2780 cells (5 × 10^6^) and Plko.1‐NC‐transfected A2780 cells (5 × 10^6^) were injected into the lateral tail veins of 6‐week‐old BALB/c nude female mice. After two months, the mice were killed under anesthesia. To assess tumor metastasis, the lungs were collected and fixed in 4% formalin. The volume (length × width2/2) and quantity (mean ± SD) of metastatic tumor were then calculated.

### 2.10 Statistical analysis

In this study, statistical analysis was carried out using SPSS 17.0 software version (Chicago, IL, USA). All cell culture experiments were independently repeated three times. The IC50 (mean ± SD) was calculated according to the cell survival curve by using Graphpad prism 5 (Graphpad Software, California, USA). The cell number of migration and cell viabilities among different groups were analyzed using unpaired two‐tailed Student's *t* test. After logarithmic transition, mRNA expression and tumor number of lung metastasis analysis were performed also using unpaired two‐tailed Student's *t* test. Chi‐square (χ^2^) test was used to measure the correlation between the CCNG1 and Notch3 expression. Overall survival (OS) and progression‐free survival (PFS) were determined using Kaplan‐Meier method. Values were represented as mean ± SD. Results obtained with *P *< 0.05 were considered to be statistically significant.

## 3 RESULTS

### 3.1 CCNG1 was overexpressed in HGSOC and associated with poor prognosis

**Figure 1:**
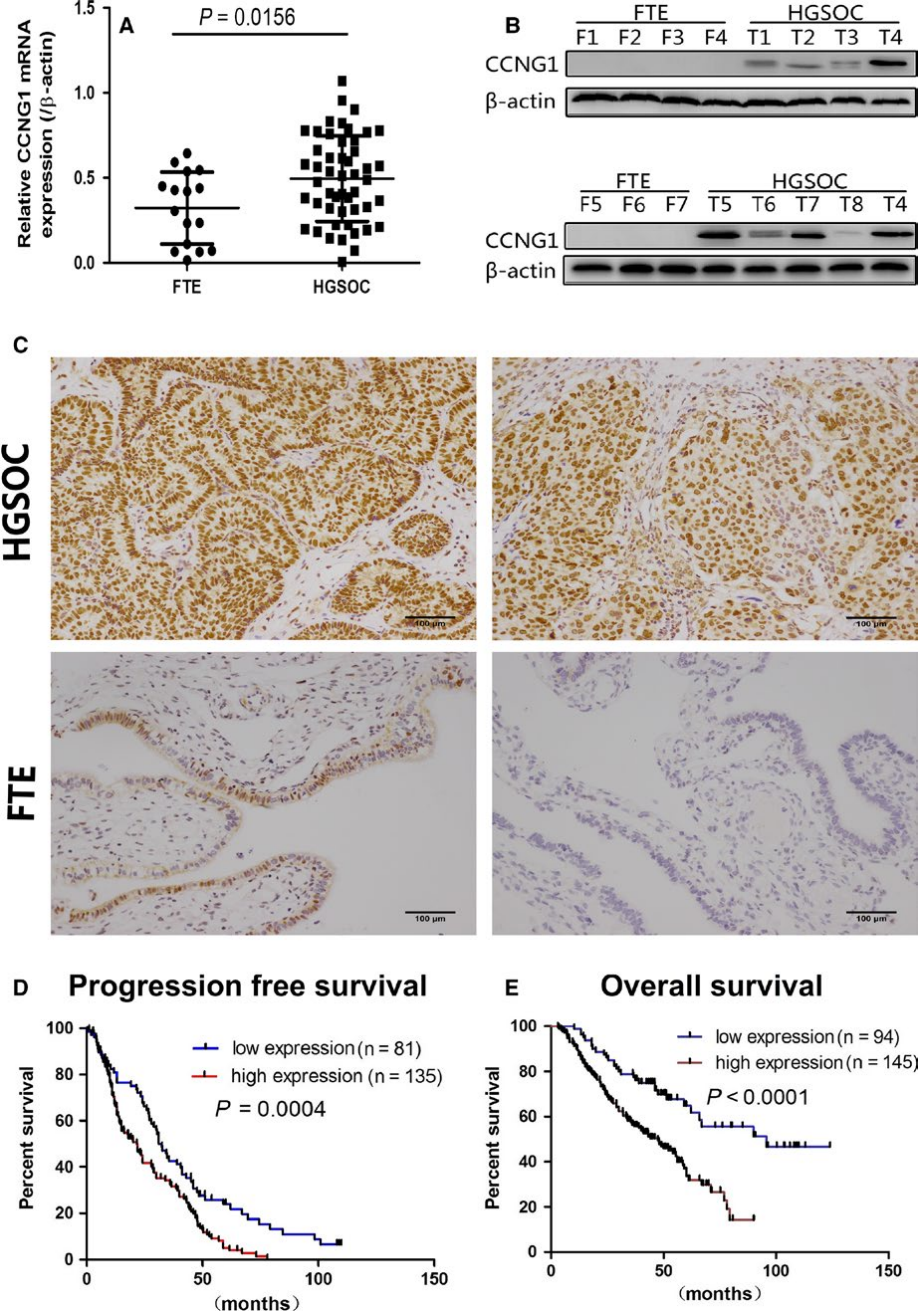
CCNG1 expression was up‐regulated in human ovarian cancer tissues. A, Relative mRNA expression (after log‐transformation) of CCNG1 in HGSOC and FTE tissues. B, WB shows protein expression of CCNG1 in HGSOC and FTE tissues. C, The expression of CCNG1 in HGSOC and FTE tissues by IHC (a&b, high CCNG1 immunoreactivity in HGSOCs; c&d, low CCNG1 immunoreactivity in FTEs). D, PFS rate of HGSOC patients with low vs. high CCNG1 expression (*P *< 0.01); E, OS rate of HGSOC patients with low vs. high CCNG1 expression (*P *< 0.01)

We analyzed the mRNA expression level of *CCNG1* in 50HGSOC tissues and 16 normal control tissues (Figure 1A) and found that the expression of CCNG1 was up‐regulated in HGSOC tissues compared to normal control tissues (*P*: 0.0156). CCNG1 protein expression was then investigated by IHC and WB (Figure 1B,C). High expression of CCNG1 was observed in HGSOC tissues (61.3%, 163/266) compared to normal control tissues (31.8%, 21/66). Additionally, high expression of CCNG1was significantly associated with a shorter OS (*P*: 0.0001) and PFS (*P*: 0.0004) (Figure 1D,E). No significant correlation between CCNG1 and other clinicopathological variables, such as age and FIGO stage, was observed (Table 4).

**Table 4:** CCNG1 expression and clinicopathological features in human high‐grade serous ovarian cancer (HGSOC) patients

Groups	n	Low expression	High expression	*P*‐value
Age (y)				
>50	89	33	56	0.790
=<50	177	70	107	
FIGO stage				
I	22	7	15	0.756
II	29	11	18	
III+IV	214	85	129	
Unknown	1	0	1	
OS (y)				
>2	176	77	99	0.0240
=<2	53	17	46	
Unknown	27	9	18	
PFS (y)				
>2	139	65	74	<0.0001
=<2	77	16	61	
Unknown	50	28	22	

### 3.2 CCNG1 induced migration and invasion of human ovarian cancer cells via promotion of epithelial‐to‐mesenchymal transition (EMT)

**Figure 2:**
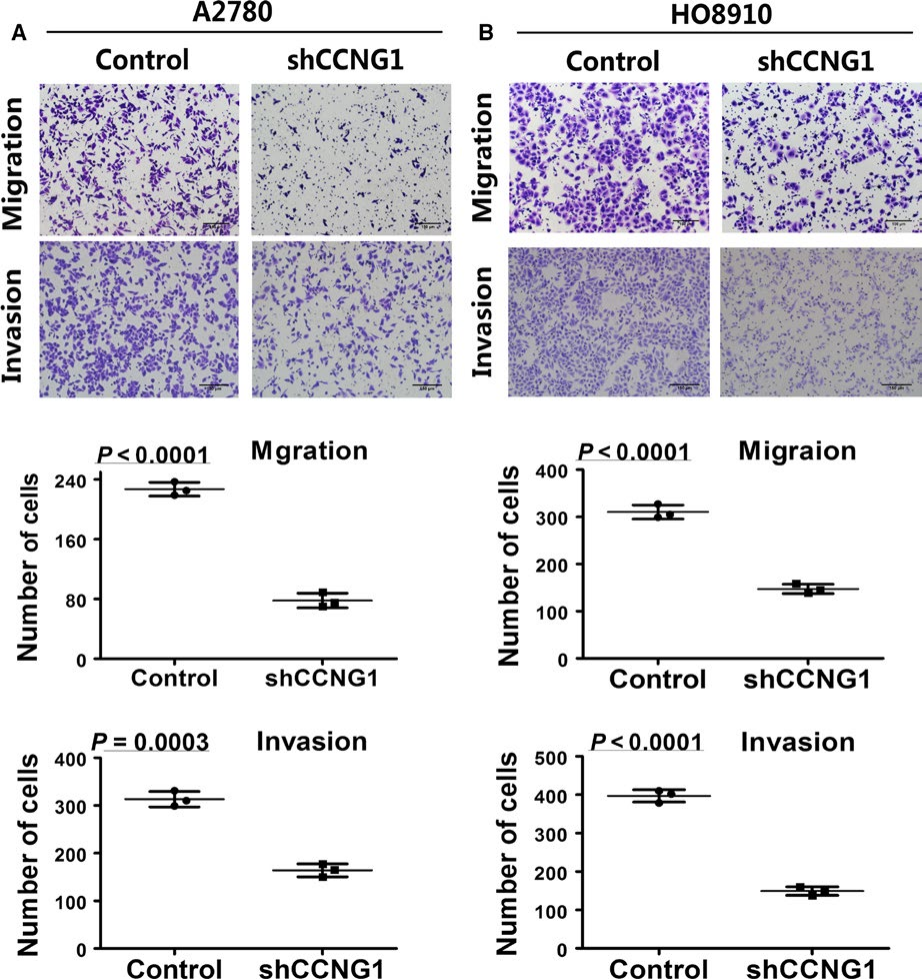
Effects of down‐regulating CCNG1 on the migration and invasion ability of ovarian cancer cell lines. The number of migrating and invasion cells in silenced CCNG1group was significantly reduced in A2780 (A) or HO8910 (B). The numerical values were mean ± SD of three replicates. All the experiments were repeated three times using the same batch of cells

**Figure 3:**
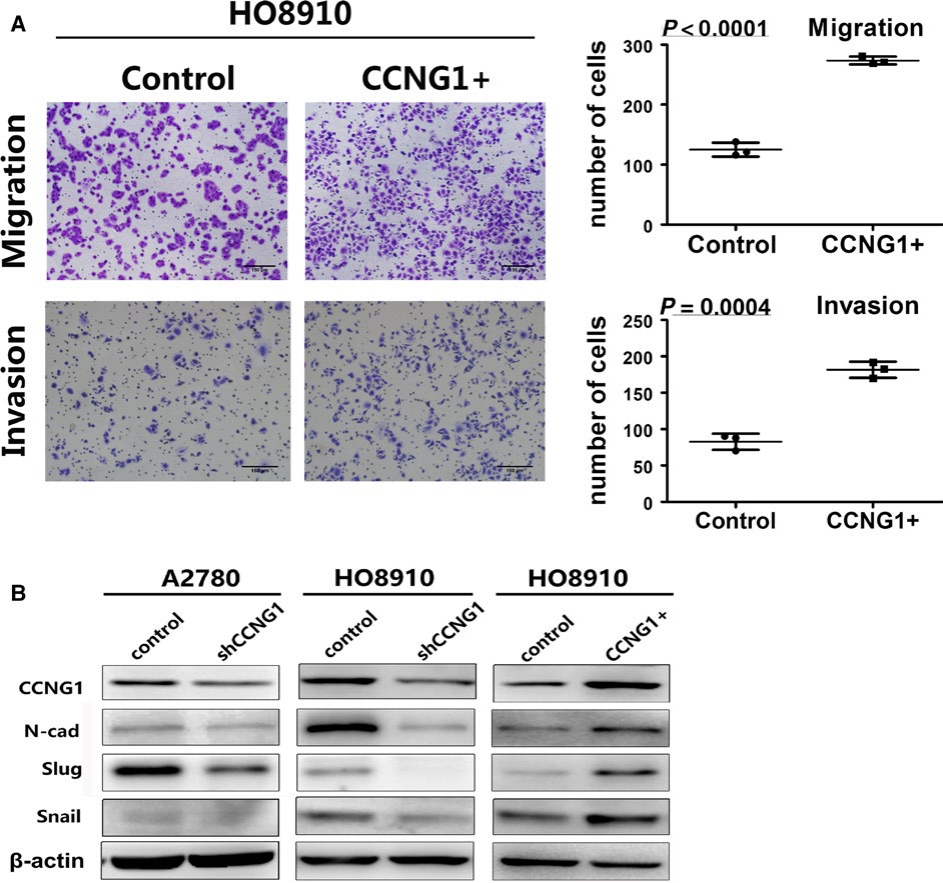
Interpretation of cell migration and invasion by regulating CCNG1 through regulating EMT. A, The number of migration and invasion cells in the CCNG1 overexpression group was increased. The numerical values were mean ± SD of three replicates. B, Extracts from the cells were analyzed for EMT marker protein N‐cad, slug, and snail expression by WB

The protein expression level of CCNG1 in ovarian cancer cell lines and normal control cell line are shown in Figure S7A. Knockdown of CCNG1 reduced ovarian cancer cells’ ability to metastasize (Figure 2A,B, *P *< 0.0001). Moreover, cells transfected with exogenous CCNG1 demonstrated significantly higher rate of metastasis (Figure 3A, *P *< 0.0001). We further investigated the underlying molecular mechanism behind this phenomenon by analyzing EMT‐related factors. We found that alteration in CCNG1 expression affected the expression of EMT‐related proteins (Figure 3B). These data suggested that CCNG1 induced cell metastasis via promotion of ovarian cancer cell EMT.

### 3.3 CCNG1 overexpression was associated with chemotherapy resistance

**Figure 4:**
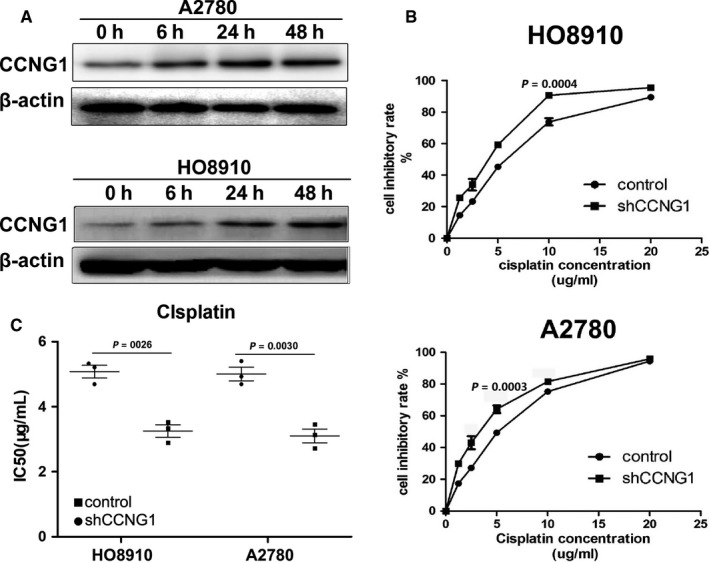
CCNG1 modulated cisplatin sensitivity. A, After exposure to cisplatin, CCNG1 protein expression in ovarian cancer cells was time‐dependently up‐regulated. B and C, Cell inhibitory rates and IC50 of cisplatin in HO8910 and A2780 were significantly changed after down‐regulation of CCNG1 expression by shRNA. Cell viability detection, from cells seeded in 96‐well plates to calculate IC50, was repeated three times to obtain three IC50

WB assay showed that there was a significant time‐dependent increase in CCNG1 expression when cells were exposed to cisplatin (Figure 4A). After inhibiting CCNG1 expression with shRNA, the half‐maximal inhibitory concentration (IC50) of HO8910 cells was significantly reduced (3.403 ± 0.385 μg/mL vs. 5.698 ± 0.354 μg/mL, *P*: 0.0026), and the cisplatin inhibition rate was increased compared to the control cells (Figure 4B,C). Similar results were seen in A2780 cells (3.234 ± 0.445 μg/mL vs 5.084 ± 0.214 μg/mL, *P*: 0.003), suggesting that CCNG1 overexpression was associated with ovarian cancer chemotherapy resistance.

### 3.4 P53mt up‐regulated CCNG1 and Notch3 expression in ovarian cancer cells

**Figure 5:**
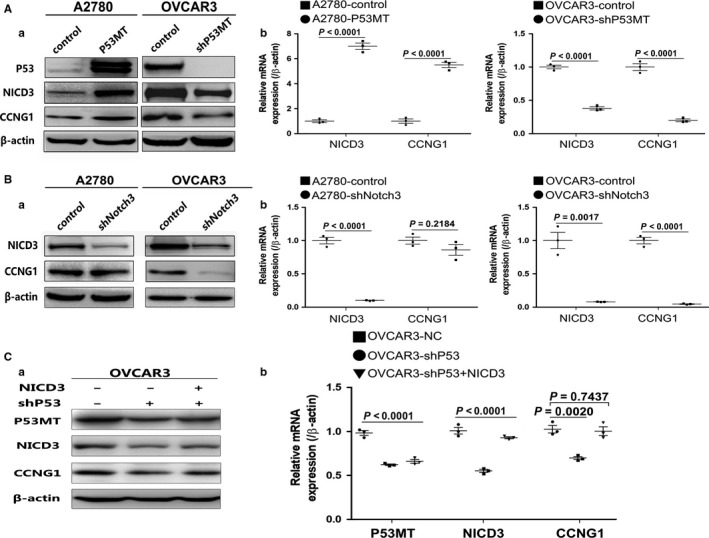
P53MT regulated CCNG1 and Notch3 expression. A, CCNG1 and NICD3 expression were both evaluated by WB(A) and RT‐PCR(B) in P53mt overexpressing cells, and CCNG1 and NICD3 protein expression evaluated in P53mt knockdown cells. B, The level of CCNG1 expression was reduced in down‐regulated Notch3. C, The down‐regulation of CCNG1 induced by knockdown of P53mt was eliminated after up‐regulation of NICD3 expression in OVCAR3 cells. Each experiment (WB, RT‐PCR) was repeated three times

It is known that P53 is wild‐type in A2780 and mutated in OVCAR3 cells. When the plasmid with ectopic P53mt135 was transfected into A2780, CCNG1 expression increased (Figure 5A and Figure S7B). In order to investigate the relationship between P53mt and Notch3, we measured the expression level of cleaved Notch3 (NICD3). We found that NICD3 was positively regulated by P53mt (Figure 5A and Figure S7B), suggesting that p53 mutation may up‐regulate CCNG1 and Notch3 expression in ovarian cancer.

### 3.5 Notch3 positively regulated CCNG1 expression in ovarian cancer

**Figure 6:**
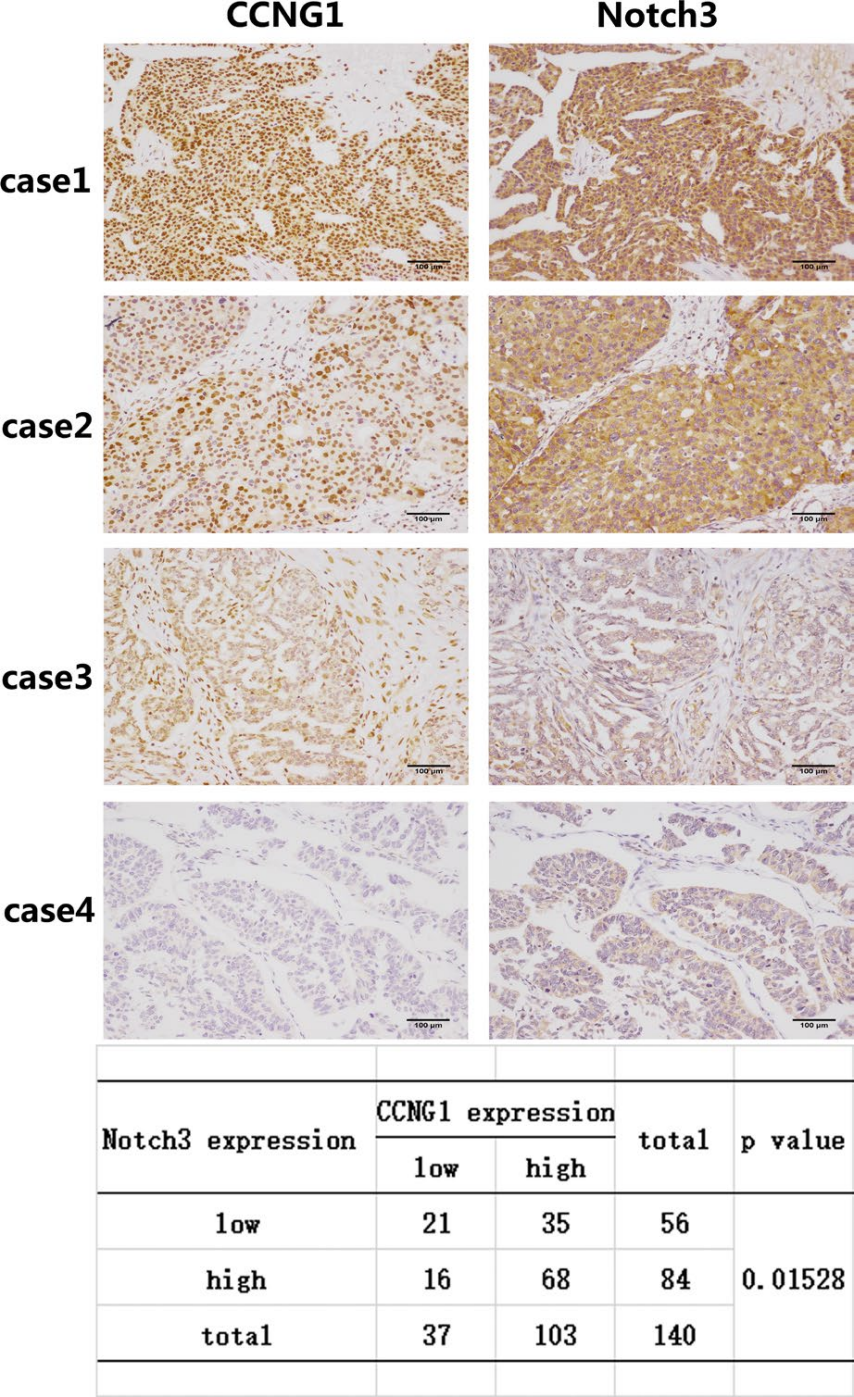
The expression of CCNG1 is associated with Notch3 expression in ovarian cancer tissue. Immunohistochemistry analysis in four representative cases showing Notch3 and CCNG1 expression in the same area. Cases 1‐2 show Notch3 and CCNG1 staining in the same area. Cases 3‐4: HGSOC tissue with Notch3 and CCNG1 negative. The expression level between Notch3 and CCNG1 was statistically significant (*P*: 0.01528). Each experiment was repeated three times

As we detected that P53mt could regulate both NICD3and CCNG1 expression in ovarian cancer cell lines, we investigated whether NICD3 expression is associated with CCNG1 expression in HGSOC. Down‐regulation of NICD3 by shRNA resulted in decreased CCNG1 expression (Figure 5B and Figure S7C), suggesting that NICD3 may be the upstream regulator of CCNG1 in ovarian cancer cells. Interestingly, in P53wt cells A2780, CCNG1 protein expression level was not changed after down‐regulating NICD3 expression (Figure 5B and Figure S7C). Furthermore, in the TMA of HGSOC, we detected a significant relationship between Notch3 and CCNG1 expression (*r* = 0.39, *P*: 0.01528) (Figure 6). These data demonstrated that Notch3 could up‐regulate CCNG1 expression and play an important role for the regulation between P53mt and CCNG1.

### 3.6 CCNG1 was regulated by P53mt through Notch3 pathway

In order to analyze the relationship between CCNG1, P53mt, and NICD3, we detected the expression of CCNG1 in shP53‐OVCAR3 cells after the up‐regulation of NICD3. As shown in Figure 5C and Figure S7D, the down‐regulation of CCNG1, caused by knockdown of P53mt, was rescued by NICD3 up‐regulation, which suggested that CCNG1 could be regulated by P53mt through the Notch3 pathway.

### 3.7 Knockdown of CCNG1 inhibited tumor metastasis of A2780 cells in vivo

**Figure 7:**
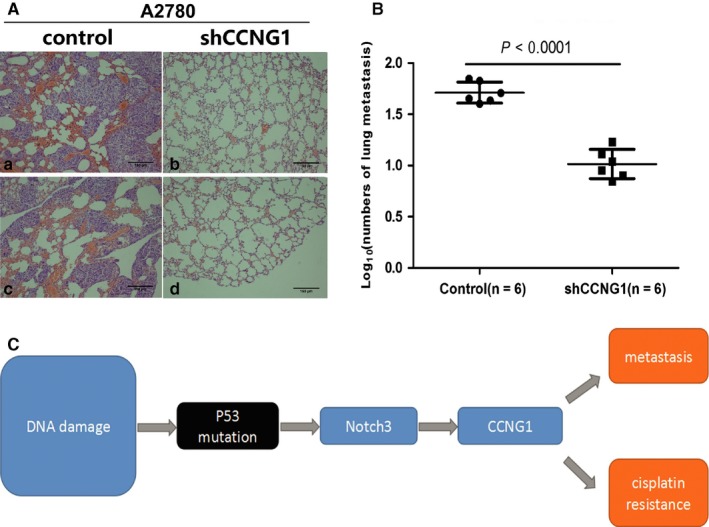
Down‐regulation of CCNG1 reduced tumor metastasis in vivo*.* A, H&E staining of lung metastasis of shCCNG1‐A2780 cell line and its control. B, Nude mice injected with shCCNG1‐A2780 cell line developed fewer lung metastatic foci than its control (*P *< 0.0001). C, CCNG1 was regulated by P53mt via induction of Notch3 expression, which ultimately promotes both HGSOC tumor cell metastasis and cisplatin resistance

ShCCNG‐A2780 cells and negative controls (n = 6) were injected into nude mice through tail veins. Seven weeks postinjection, lung tissue was procured to assess for metastasis (Figure 7A). As expected, the lungs from mice injected with shCCNG1‐A2780 cells developed fewer metastatic foci than the control group (Figure 7B). These data supported the idea that CCNG1 promotes ovarian cancer metastasis.

## 4 DISCUSSION

Ovarian cancer is the fifth‐leading cause of cancer death among women in the United States,^1^, ^5^, ^24^, ^25^ and HGSOC is the major type often with poor patient outcomes. There are several reasons for the poor prognosis of HGSOC. First, most patients with HGSOC are diagnosed at an advanced stage due to initial asymptomatic clinical course and high tumor metastasis rate. Second, although HGSOC patients are initially extremely sensitive to platinum‐based chemotherapy, they usually relapse being characterized by acquisition of chemotherapy resistance.^26^ In this regard, understanding the molecular mechanism underlying HGSOC metastasis and chemotherapy resistance may provide novel therapeutic options in improving clinical outcomes for patients with HGSOC. For decades, great efforts have been made to elucidate the molecular mechanism underlying tumorigenesis, invasion, and metastasis of HGSOC; however, the detailed mechanism of HGSOC progression remains obscure.

In this study, we found that CCNG1 expression was correlated with a poor prognosis in HGSOC patients. In vitro and in vivo experiments demonstrated that CCNG1 promoted EMT and facilitated metastasis of ovarian cancer cells. Additionally, as evidenced by our data, CCNG1 overexpression reduced the sensitivity of ovarian cancer cells to cisplatin and was associated with a shorter survival, and *vice versa*. Our data highlighted the potential enhancement of chemotherapy resistance in HGSOC induced by CCNG1 overexpression.

Recent evidencerevealed an important role of Notch signaling in HGSOC cancer progression. Notch pathway alterations are present in roughly 23% of HGSOC and that its dysregulation is associated with poor overall survival.^21^ Our data suggested that there might be an association between P53mt and Notch3 in ovarian cancer development. Previous studies show that P53wt indirectly inhibits Notch transcriptional activity and, in turn, Notch acting as an oncogene inhibits P53 activity in several types of cancers.^27^, ^28^ It remains unclear, however, how the P53mt phenotype affects Notch protein expression. We found in our study that P53mt up‐regulated the expression of Notch3, suggesting that P53mt serves a regulatory role in the Notch3 pathway.

Kimuraet et al shown that CCNG1 could promote TP53 degradation through the MDM2 pathway. The dysregulation of CCNG1 expression is associated with genomic instability and DNA damage.^29^, ^30^ CCNG1 expression may also be down‐regulated by MDM2 through proteasome‐mediated degradation as a part of a negative feedback loop in the P53‐MDM2‐CCNG1 pathway.^29^, ^31^, ^32^ In our study, we primarily found that CCNG1 could be positively regulated by P53mt and Notch3 in HGSOC. As shown in our results, there was a resultant decrease in CCNG1 expression after inhibiting Notch3 expression either with or without P53mt. This finding indicates that Notch3 is required for regulating CCNG1 protein expression in P53mt cells. Moreover, a strong correlation between Notch3 and CCNG1 expression levels supports a role of both Notch3 and CCNG1 in cancer progression. These data overall reveal a molecular mechanism that provides a plausible explanation for promotion of metastasis and cisplatin resistance by P53mt‐Notch3 pathway via up‐regulation of CCNG1 expression (Figure 7C).

In summary, our findings indicate that CCNG1 overexpression, which is associated with poor clinical prognosis of HGSOC, can promote metastasis and chemotherapy resistance via the P53mt‐Notch3 pathway. Our data also suggest that CCNG1 inhibitors represent a novel approach to increase chemotherapy efficacy in HGSOC and potentially improve patient outcomes.

## 5 ETHICS STATEMENT

The current study was approved by the Qilu Hospital of Shandong University's Institutional Review Board (Jinan, China). The patient samples from the participating institutions included in this study were obtained with signed informed consent in accordance with ethics committee requirements.

## CONFLICT OF INTEREST

None declared.
