# Supplementary figures and images for: CCNG1 (Cyclin G1) regulation by mutant‐P53 via induction of Notch3 expression promotes high‐grade serous ovarian cancer (HGSOC) tumorigenesis and progression
Source: Cancer Med. 2018 Dec 18;8(1):351–62. doi: 10.1002/cam4.1812 (PMC6346265; doi:10.1002/cam4.1812)

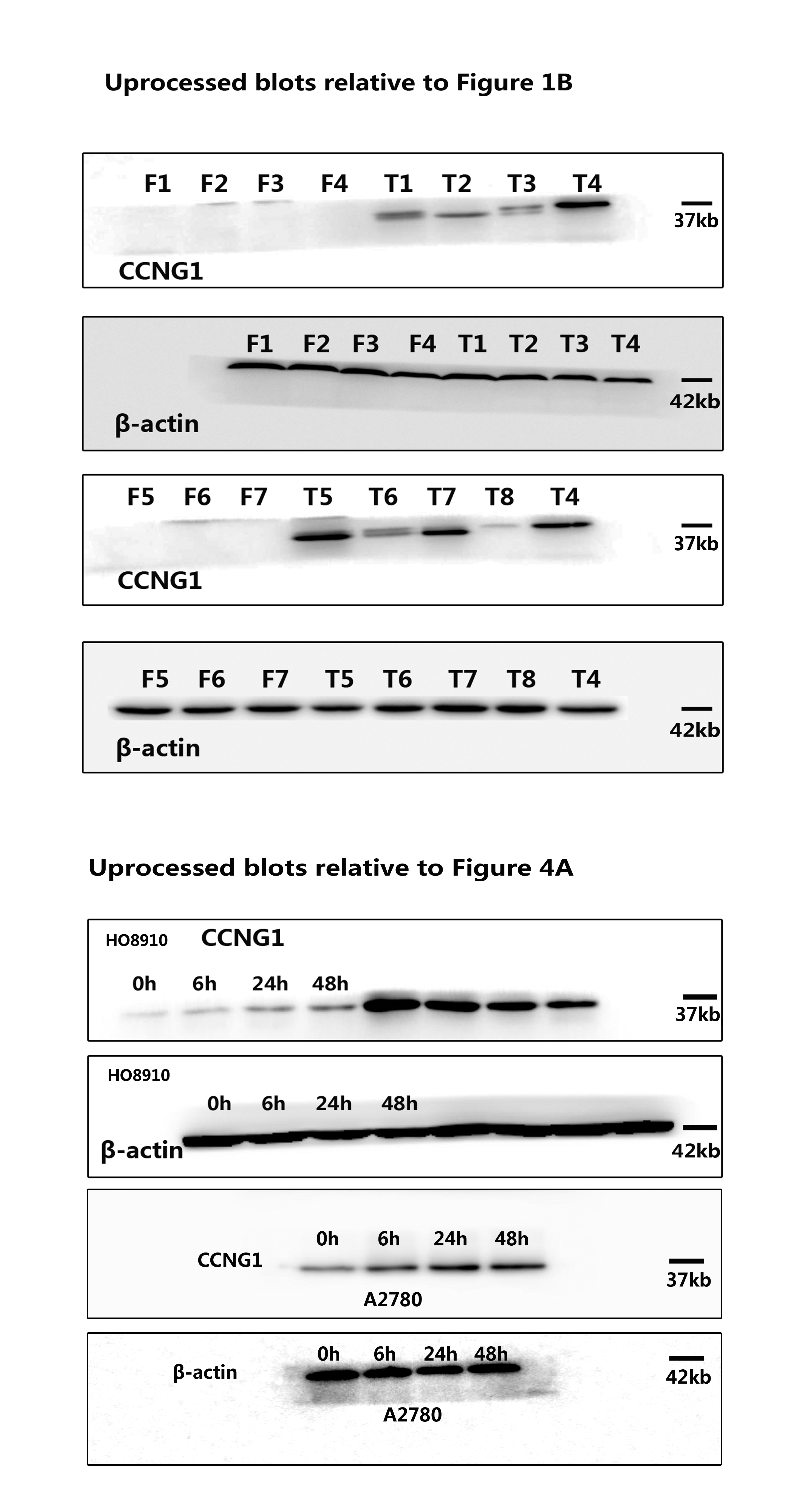

Supplement: Supplementary file 1 [file CAM4-8-351-s001.tif]

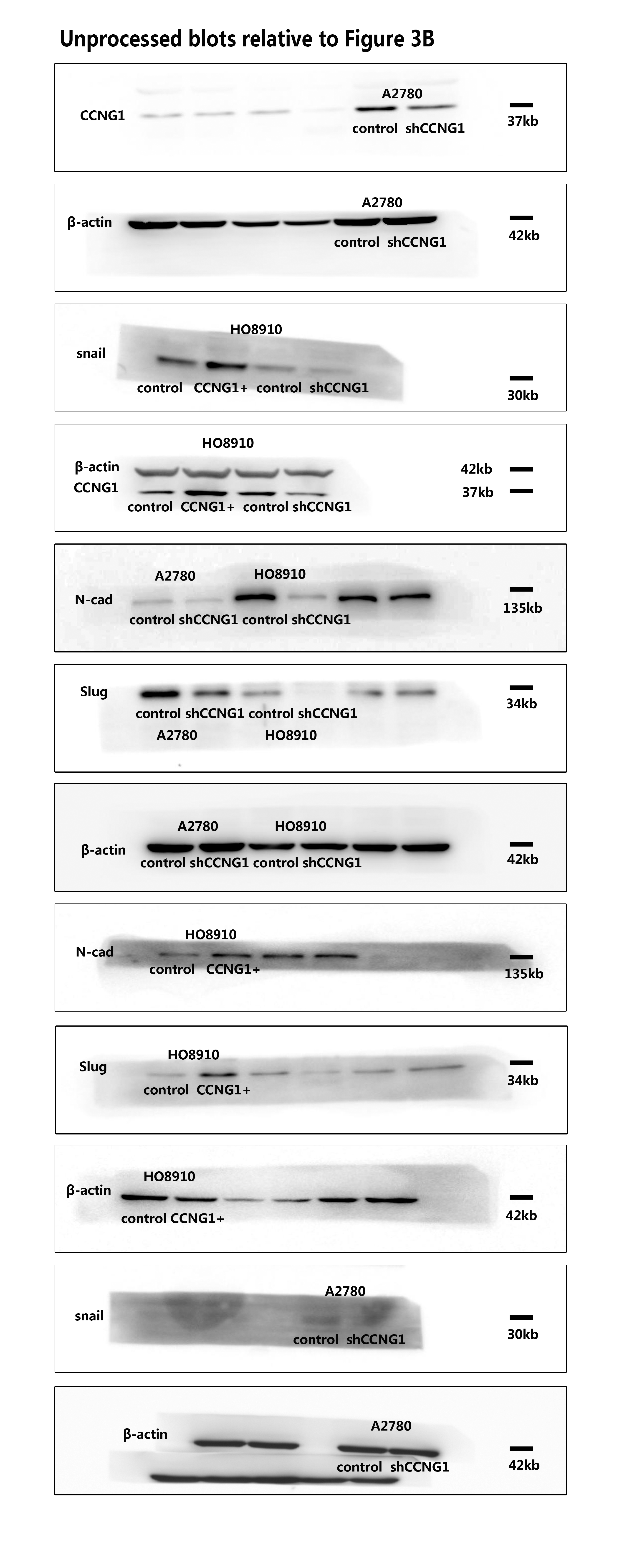

Supplement: Supplementary file 2 [file CAM4-8-351-s002.tif]

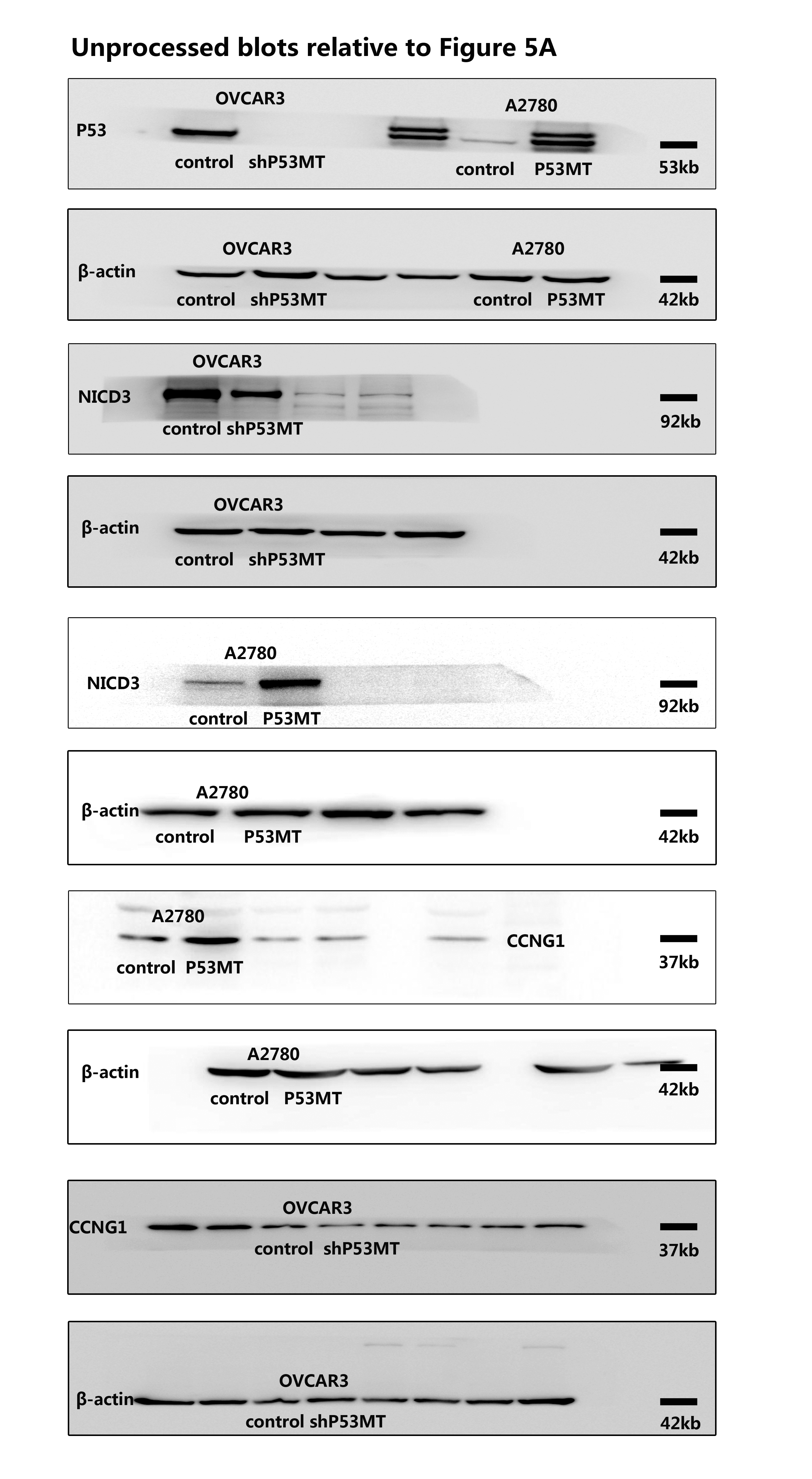

Supplement: Supplementary file 3 [file CAM4-8-351-s003.tif]

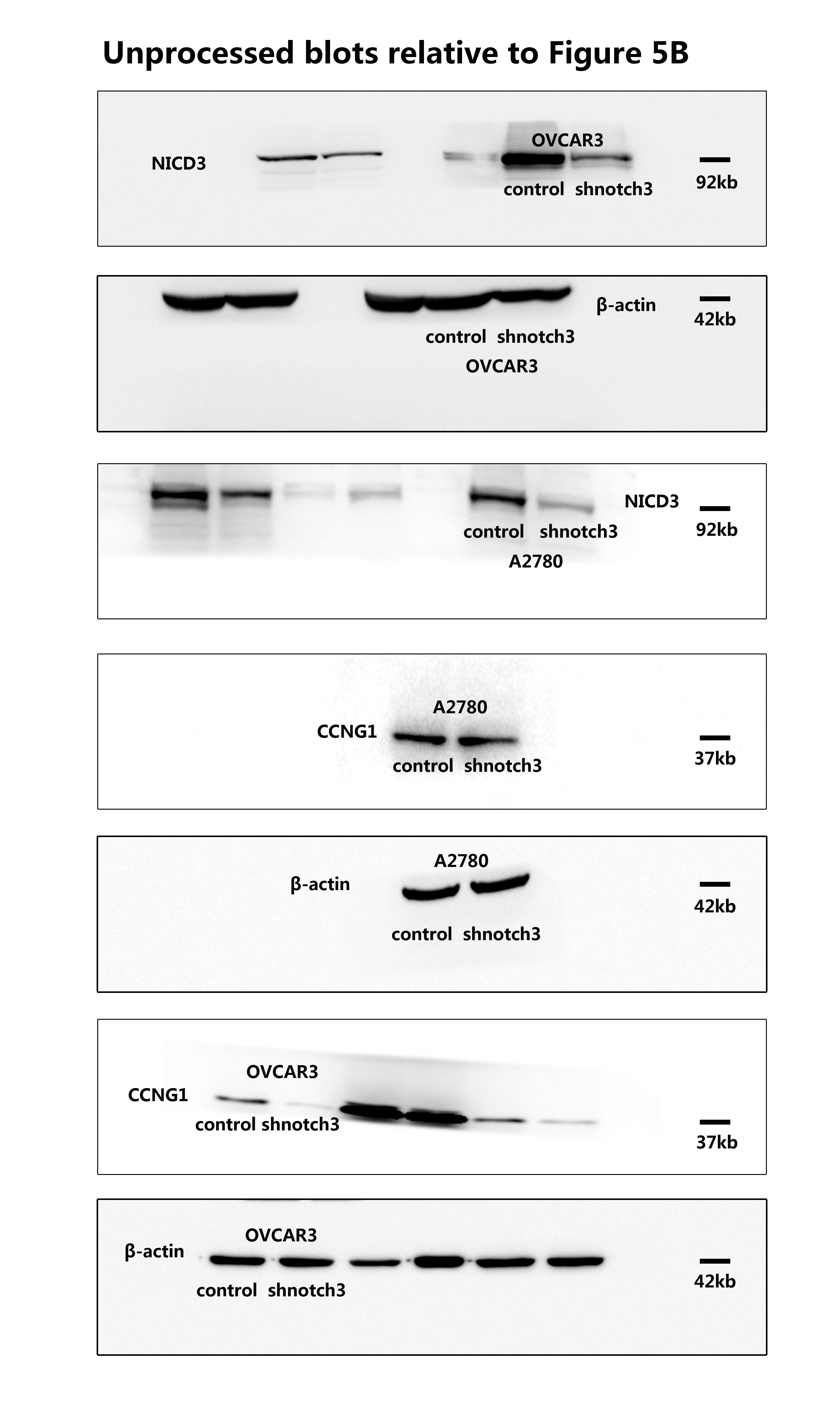

Supplement: Supplementary file 4 [file CAM4-8-351-s004.tif]

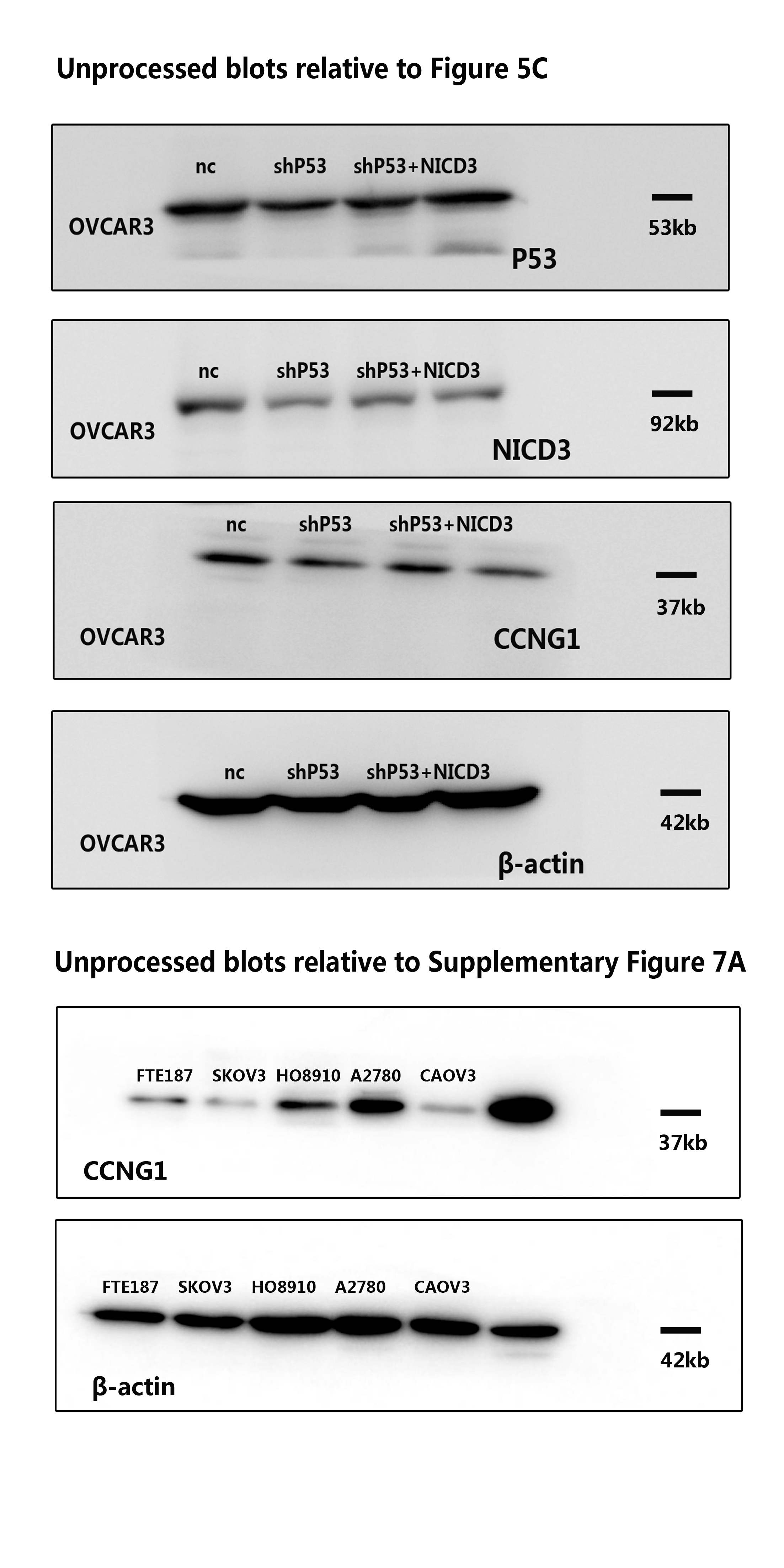

Supplement: Supplementary file 5 [file CAM4-8-351-s005.tif]

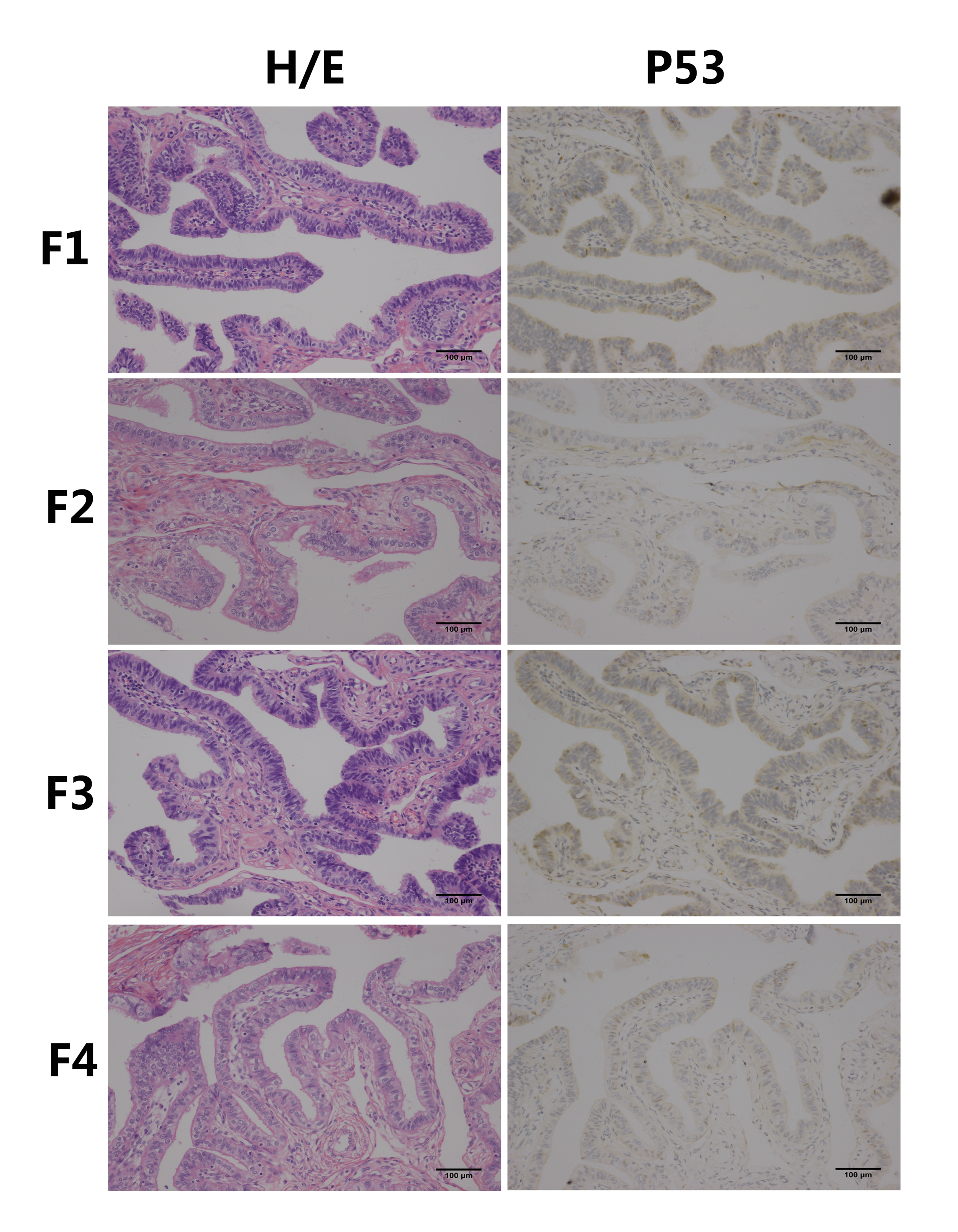

Supplement: Supplementary file 6 [file CAM4-8-351-s006.tif]

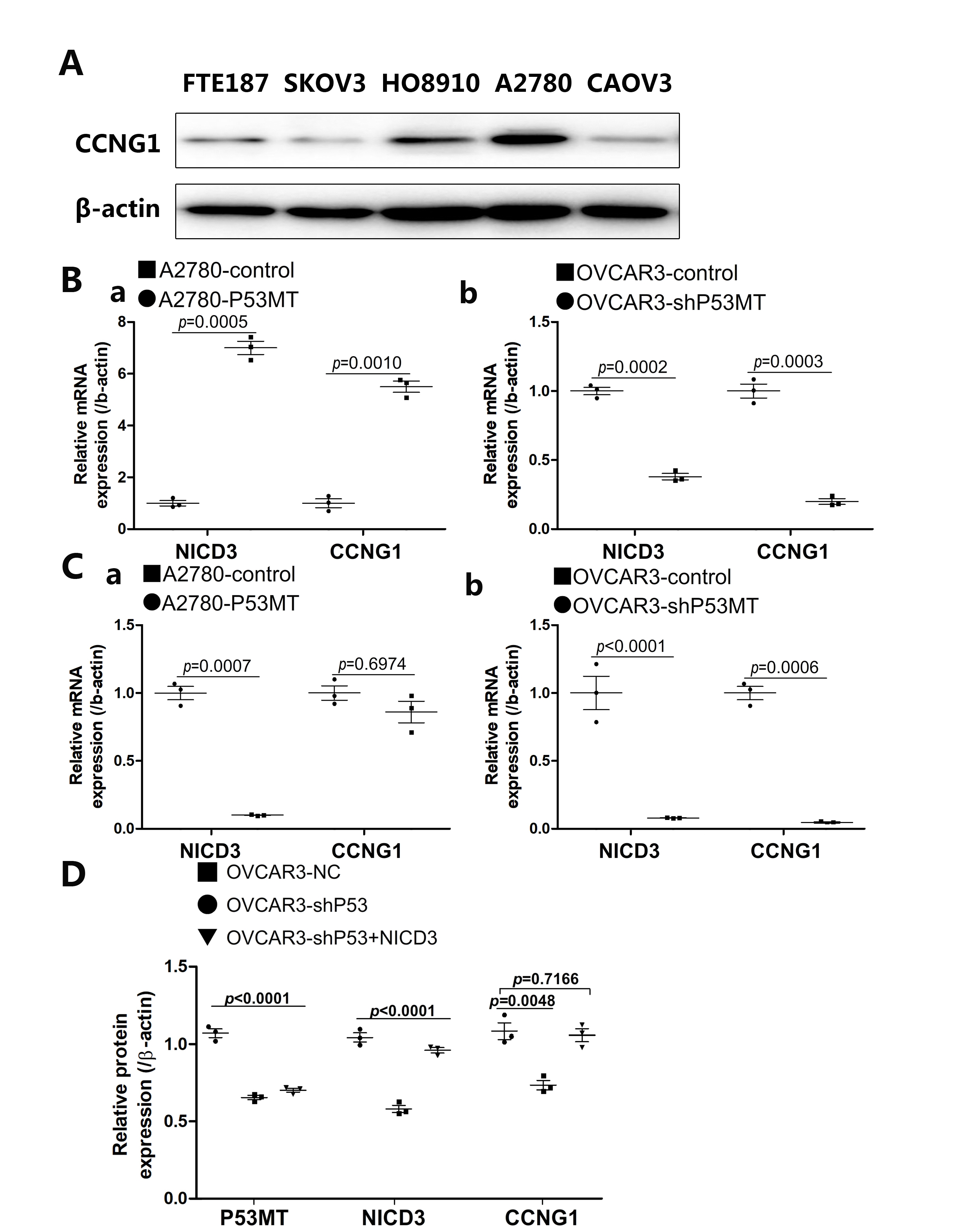

Supplement: Supplementary file 7 [file CAM4-8-351-s007.tif]
