# Supplementary material for: CCNG1 (Cyclin G1) regulation by mutant‐P53 via induction of Notch3 expression promotes high‐grade serous ovarian cancer (HGSOC) tumorigenesis and progression
Source: Cancer Med. 2018 Dec 18;8(1):351–62. doi: 10.1002/cam4.1812 (PMC6346265; doi:10.1002/cam4.1812)
Supplement: Supplementary file 8 [file CAM4-8-351-s008.docx]

Appendix S1

1. Seed HEK293T cells to be 70% confluent at transfection in 10cm culture dish.
2. Dilute 36μL Lipofectamine® 2000 Reagent in 1.5mL Opti-MEM Medium.
3. Dilute 8μg psPAX2 vector, 4μg pMD2G vector and 8μg target gene vector in 1.5mL Opti-MEM Medium.
4. Add diluted vector (step 4) to diluted Lipofectamine® 2000 Reagent (step 5).
5. Incubate for 10 mins.
6. Add vector-lipid complex to cells, and incubate for 3 hours.
7. 6ml Opti-MEM Medium with 10% FBS is exchanged to vector-lipid complex.
8. After 24 hours, the Lentivirus is harvest.
